# Molecular mechanisms and mode of tamoxifen resistance in breast cancer

**DOI:** 10.6026/97320630012135

**Published:** 2016-06-15

**Authors:** Shazia Ali, Mahmood Rasool, Hani Chaoudhry, Peter N Pushparaj, Prakash Jha, Abdul Hafiz, Maryam Mahfooz, Ghufrana Abdus Sami, Mohammad Azhar Kamal, Sania Bashir, Ashraf Ali, Mohammad Sarwar Jamal

**Affiliations:** 1 School of life science, Jawaharlal Nehru University, New Delhi, India;; 2 Center of Excellence in Genomic Medicine Research, King Abdulaziz University, Jeddah, Saudi Arabia;; 3 Department of Biochemistry, Faculty of Science, Center of Innovation in Personalized Medicine, King Fahd Center for Medical Research, King Abdulaziz University, Jeddah, Saudi Arabia;; 4 Department of Computer Science, Jamia Millia Islamia, New Delhi, India;; 5 Ummul Qura University, Makkah, Saudi Arabia;; 6 Department of Biotechnology, Jamia Millia Islamia, New Delhi, India;; 7 King Fahd Medical Research Center, King Abdulaziz University, Jeddah, Kingdom of Saudi Arabia;

**Keywords:** Breast carcinoma, Estrogen receptor, Tamoxifen resistance, Endocrine therapy

## Abstract

Breast cancer is one of the most common cancers in women around the globe Tamoxifen is used for the last 40 years as an endocrine therapy for breast cancer. This resulted in the reduction of mortality rate by 30% and it still remains one of the most effective therapies against breast cancer. However, resistance against tamoxifen is still one of the major hurdles in the effective management of breast cancer. Intense research has been conducted in the past decade to further explore its resistance mechanism, but still a lot of research will be needed to effectively alleviate this problem. Several biochemical factors and molecular pathways, such as the modulation of ER signaling, upregulation of growth factors had been observed as key factors for tamoxifen resistance (TR). After, initial therapy of five to ten years, breast cancer patients develops resistance towards this drug. The resistance leads to the development of other cancers like uterine cancer. Here, we briefly explore all the molecular events related to tamoxifen resistance and focus on its mechanism of action as well as other pharmacological approaches to better its beneficial effects in the treatment of breast carcinoma.

## Background

**Figure 1:**
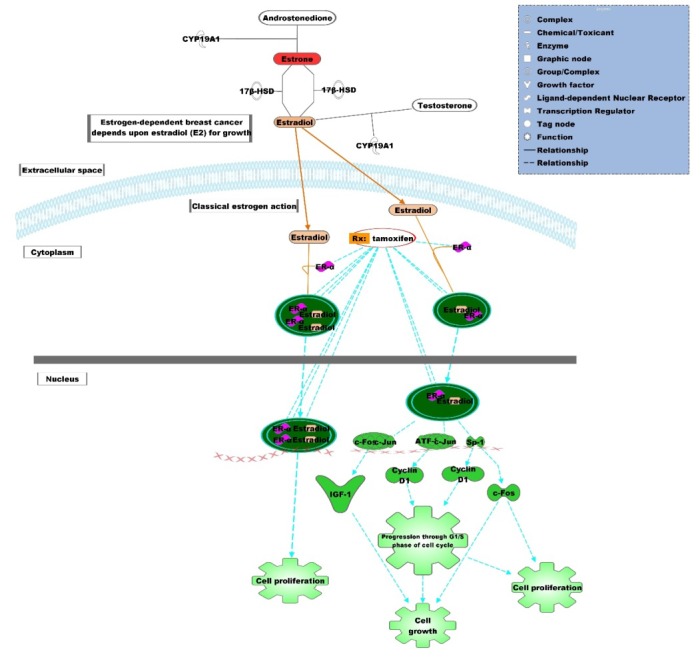
Competitive Binding of Estrogen and Tamoxifen to Estrogen Receptor in Breast Cancer.The binding of estrogen with estrogen receptor is displaced by tamoxifen which binds to estrogen receptor and inhibit cell proliferation. The molecular mechanism of Tamoxifen-mediated inhibition of cell growth and cell proliferation in estrogen-induced breast cancer was obtained using Ingenuity Pathway Analysis (Qiagen, USA).

Breast cancer is one of the major malignancies affecting women across the world. In 2012 around 1.7 million people were diagnosed with this malignancy and about 522,000 deaths were reported in the same year [1]. About 70% of breast carcinoma expresses the α-estrogen receptor (ER+). ER plays a major role in the tumorigenesis of breast cancer as it upregulates cyclin D1, Myc, Bcl-2 and VEGF (Vascular endothelial growth factor), which play a significant role in cell cycle, cell survival and the stimulation of angiogenesis [2]. For most of the ER+ patients, endocrine therapy is the first choice for treatment. Presently, three classes of endocrine therapies are widely used , namely, selective estrogen receptor modifiers (SERMs) such as tamoxifen which blocks transcriptional activity of ER by directly binding to it, selective estrogen receptor down regulators (SERDs), such as fulvestrant, and aromatase inhibitors (AIs) such as lentrozole, anastrozole and exemestane that inhibit aromatase enzyme responsible for estrogen production [3]. However, Tamoxifen has been the first choice for adjuvant therapy since its discovery in 1970, particularly for premenopausal women as it reduces breast cancer recurrence and annual mortality rate by 50% and 31% respectively. Despite this success, 20-30% tumors are resistant to tamoxifen therapy, which was either present before the treatment (de-novo resistance) or develop resistance during the therapy (acquired tamoxifen resistance). There are several factors exists, which is speculated to be responsible of tamoxifen resistance. Among these factors, alteration of ER signaling, crosstalk between ER and GFR (growth factor receptor) network, downregulation of ER, upregulation of specific GFR, activation of PI3/AKT/mTOR pathway including PTEN inactivation and induction of NFκB signaling are prominent factors leading to tamoxifen resistance [4]. Breast cancer stem cells (BCSCs) are responsible for tumor evolution and resistance to tamoxifen [5]. There is no dearth of articles explaining breast cancer and tamoxifen resistance. Here, we have given an overview of the mechanism of evolution of tamoxifen resistance. Tamoxifen is a selective estrogen modulator that competes with the estrogen for estrogen receptor and displaces estrogen and thereby inhibits estrogen function in breast tissue rather than suppression of circulating estrogen levels (Figure 1). Tamoxifen binding does not change the receptor’s shape in same manner as estrogen binding. The co-activators are not binding but, inhibiting the activation of genes that enhance cell proliferation, thereby it inhibits the function of estrogen to increase the proliferation of breast cells. The estrogen receptor and tamoxifen complex recruits different proteins present in the cell as co-repressors such as NCoR (nuclear receptor co-repressor) and SMRT proteins which represses the gene stimulation [6]. NCoR is a transcriptional coregulatory protein which is also known as thyroid hormone and retinoic acid receptor associated co-repressor 1 (TRAC-1). In humans this protein is encoded by NCOR1 gene. This protein contains several nuclear receptor interacting domains and also recruits histone deacetylase to DNA promoter regions, assisting down regulation of gene expression. NcoR and SMRT binds directly to transcription factors and form stable complexes with histone deacetylase 3, transducin b like protein1/TBL1 related protein1 and G protein pathway receptor, and participate in the deregulation of transcription factors [7].

Tamoxifen is used as an endocrine therapy for estrogen receptor positive breast cancer in pre-menopausal and post-menopausal women for last 40 years. It is given with aromatase inhibitors, for example, in postmenopausal women, anastrozole and letrozole as adjuvant therapy. Besides, it is also used in the treatment of male breast cancer [8]. Exemestane is also given to early-stage postmenopausal women having breast cancer. It reduces recurrence, number of deaths and symptom in other breast cancer patients. It lowers risk of breast cancer in lobular carcinoma.

There are about 50% estrogen receptor positive breast cancers which are not cured by endocrine therapies due to intrinsic resistance. Tamoxifen given to the rest of the women also develop resistance after 3-5 years of its intake. Tamoxifen acts as an agonist in endometrium and over a period of time, tamoxifen intake results in endometrial cancer. About 30% of women having primary stage breast cancer suffer from recurrence of the disease [9]. Tamoxifen function is regulated in a cell by various growth factors, for example, growth factor proteins ErbB2/HER2 are blocked as they occur in high levels in tamoxifen resistant cancers.

### Metabolism of tamoxifen

Tamoxifen is a prodrug and has little affinity for its target protein called as estrogen receptor. It is metabolized in liver by cytochrome P450-CYP26 and CYP3A4 isoforms into functional metabolites like 4-hydroxy tamoxifen (afimoxifene) and Ndesmethyl- 4-1hydroxy tamoxifen (endoxifen), which bind more effectively to its target protein estrogen receptor than tamoxifen itself, for example, 4-hydroxytamoxifen inhibits the transcription of estrogen responsive genes by functioning as an estrogen receptor antagonist in breast tissue [10, 11,12]. After binding 4- hydroxytamoxifen with ER, the ER/tamoxifen complex brings co-repressor proteins such as NCoR and SMRT which regulates the functions of several genes [7,13]. Tamoxifen needs another protein PAX2 to execute its anticancer effect as it helps in the suppression of pro-proliferative protein ERBB2 [14]. It has been found that tamoxifen effects vary with dose and response in breast cancer patients. In rats using dimethylbenzanthracene (DMBA), breast tumors were induced to study the anti-tumor function of tamoxifen in vivo. In DMBA-rat mammary carcinoma model, it has been shown that large doses of tamoxifen when given daily, completely prevented the development of tumors with short period of therapy. However, continuous therapy with small doses given daily resulted in 80% of the animals without tumor development. The adjuvant tamoxifen therapy is more beneficial if given for longer period of time, than small duration in the estrogen-receptor-positive patients. The anti-estrogens were more effective in controlling tumorigenesis, by continuous therapy for longer time period. In some cases, tumors appear again, they are then treated by anti-hormone therapy in which estrogen as well as ovaries is removed. In the adjuvant tamoxifen therapy, the outcome was that longer time period in therapy was better and, this also paved a way to use of aromatase inhibitor along with tamoxifen. The benefit of tamoxifen was its less serious side effects. With long term therapy, some of the side effects appear in breast cancer patients, for example, osteoporosis or the risk of coronary heart disease (CHD) in women are the two common side effects of tamoxifen [15]. Interestingly, tamoxifen is anti-estrogenic in breast and mammary tissue but, functions as an estrogen in bone and lowers circulating cholesterol in the body; tamoxifen has also been used for hypercholesterolemia [16,17]. The common dose of tamoxifen is 20mg/day, if it is reduced and given in transcutaneous mode, it will have less side effects in breast cancer patients. Tamoxifen metabolite, 4- hydroxytamoxifen does not cause systemic toxicity as compared to tamoxifen. There is a 16 to 18 fold variation in dose-response of these drugs in breast cancer patients [9]. Cytochrome P450 Polymorphisms that are correlated with the action of different metabolites of tamoxifen gives an explanation about the different gene variables in CYP2D6 gene and the mode of action that inhibits the function of this gene leads to toxic effects [9, 12,18].

### Mechanism of tamoxifen resistance

The resistance of tamoxifen can be explored by pharmacological studies, change in the structure, abnormal expression of micro RNAs and function of ER in tumor microenvironment and genetic changes associated with it. The expression of estrogen receptor upon binding to tamoxifen also shows how the resistance to tamoxifen will develop in the tumor microenvironment.The alteration in the expression of ERα or ERβ, change in co-regulatory proteins, abnormal expression of micro RNA, and genetic polymorphisms play a role in tamoxifen metabolic activity [9,19]. Intensive research has been done to decipher the mechanism of tamoxifen resistance, resulting in the identification of complex pathways including modulation of ER signaling, up regulation of growth factor receptors (HER2, EGFR, FGFR, IGFIR), alterations of the PI3K-PTEN/AKT/mTOR pathway and NFkB signaling [4]. Various key molecular pathways have been implicated with tamoxifen resistance such as mitogen activated protein kinases (MAPK), protein kinase A,and PAK-1 which induces the phosphorylation of estrogen receptors or its co-regulatory molecules [5,19, 20]. There are various signaling pathways and molecules involved in tamoxifen resistance. The earlier studies showed that when HER2/neu and A1B1 is over expressed, breast tumor is resistant to tamoxifen therapy through genetic changes, while in acquired resistance to tamoxifen, it is not the case [21]. Several miRNAs has been implicated in various cancers including breast cancer tumorigenesis. The expression of miRs such as miR-101, miR-206 and miR-221/miR222 has been shown to provide resistance to tamoxifen in ER positive MCF-7 cells [22]. Specific SNPs of CYP2D6 is responsible for null or reduced enzyme activity leading to poor response against tamoxifen [12].

Acquired tamoxifen resistance is the major limitation in the efficacy of tamoxifen in 50% of ER+ breast cancers. In some cases, it has been seen observed that the resistance causes tumors to be hormone independent, even if estrogen receptor is present while, as in other tumors, it shifts from estrogen receptor positive to negative.. Estrogen receptor is expressed in two third of tumors having resistance to tamoxifen but, it is inhibited when second line of hormonal therapy is initiated. The progression of disease leading to stimulation of tumor growth is called as withdrawal response. The possible reason for this withdrawal is, due to variant estrogen receptors, altered expression of other transcription factors which interact with estrogen receptors changing its confirmation. This leads to either activate or block various signal transduction pathways [23]. The antiestrogen treatment fails if the balance is not maintained between proliferation and apoptosis, for example, if growth signals are stimulated, the apoptotic signals are inhibited. The anti-apoptotic genes are increased by estrogen, protecting a cell from death inducing signals. The pro-apoptotic genes are decreased and antiapoptotic genes are increased by continuous use of tamoxifen treatment. The result is that both estrogen and tamoxifen cause increase in anti-apoptotic genes involved in tumor homeostasis, which impairs normal growth in a cell. Impaired regulation of anti-apoptotic Bcl-2 family members such as Bcl-2, Bcl-xL, and MCL-1 are implicated in the development of various cancers including breast cancer. The tamoxifen resistance is caused by estrogen via promoting Bcl-2: Bax ratio by estrogen. Besides, HER-2 overexpression increases the anti-apoptotic Bcl-2 and BclxL proteins which leads to the reduction in tamoxifen induced apoptosis and boosts tamoxifen resistance [20]. In an important study it was found that apoptosis was inducedby tamoxifen through protein phosphatase 2A–dependent phospho-Akt inactivation in estrogen receptor– negative human breast cancer cells [24]. Tamoxifen can cause apoptosis in dose and time dependent manner by regulating bcl-2 in breast cancer cells but independent of alteration in the p53 levels [25].

### Methods to overcome tamoxifen resistance

Understanding the mechanism of tamoxifen resistance shows the basis for developing drugs which target interconnected pathways. Clinical trials with exciting results are ongoing to observe the mixtures of endocrine agents with or without these drugs. Co-targeting of ReceptorTyrosine Kinase (RTK) molecular pathways and intracellular signaling networks will be one of the most promising anti-cancer approaches. Drugs targeting other signaling pathways such as PI3K-mTOR–Akt axis are currently under development. Furthermore, treating tumors with specific ER mutations with robust anti-estrogen drugs is also a fascinating approach. There are other approaches to combat tamoxifen resistance. One way is to target cell cycle proteins along with SERMs, for example, by the use of CDK-4/6 and histone deacetylase inhibitors in combination with tamoxifen, inhibiting cell cycle progression [4,14]. Another approach is to target AKT pathway by these histone deacetylase inhibitors, which have been approved by the U.S. Food and Drug Administration (FDA) for treating a rare type of lymphoma, as AKT helps in proliferation of cells in normal condition but in breast cancer it maybe be elevated and allows cancer cells to use ER in the presence of tamoxifen [26]. It has been found that MYC protein is highly expressed in cancer cells because of its interaction with HOXB7 with estrogen receptors and from cancer database patients who have high level of this gene have poor survival rates as compared to lesser ones [26]. Targeting of this pathway by MYC inhibitors from start, can ward off cancer cells and reduces the recurrence by tamoxifen resistance [27]. It has been shown that active tamoxifen metabolite endoxifen is used for early treatment of breast cancer and it has been found by clinical trials that women who are not cured better by tamoxifen and aromatase inhibitors can be treated by endoxifen.

## Conclusion:

Tamoxifen resistance is a major challenge in breast cancer therapy. Exploring the mechanisms responsible for tamoxifen resistance is essential to develop next generation of targeted therapies against breast cancer. Cutting edge research in the past have identified several factors responsible for tamoxifen resistance including crosstalk between ER and a set of growth factors [HER], impaired activation of PI3K/PTEN and NFKB activation. Targeting these pathways can provide clues to solve the issue of tamoxifen resistance in the future. Importantly, the use of tamoxifen for breast cancer treatment can further be improved by pharmacological studies which may add benefits to people suffering from cancer. The anti-cancer drugs are given either in high doses or based on population studies and the patients are not assessed for personalized dosage which results in side effects and low efficacy that eventually results in poor treatment outcomes in patients. The pharmacological and genetic basis that decides the nature and mode of action of chemotherapy is in need of both standardization of dosage and response to tamoxifen. The use of tamoxifen for breast carcinoma can be validated with more clinical trials involving large number of patients to obtain the optimized dose with less toxicity for bettering the treatment of breast cancer.
